# Evidence for a role of dystroglycan regulating the membrane architecture of astroglial endfeet

**DOI:** 10.1111/j.1460-9568.2011.07688.x

**Published:** 2011-06

**Authors:** Susan Noell, Karen Wolburg-Buchholz, Andreas F Mack, Aaron M Beedle, Jakob S Satz, Kevin P Campbell, Hartwig Wolburg, Petra Fallier-Becker

**Affiliations:** 1 Department of Neurosurgery, Tübingen Medical SchoolTübingen, Germany; 2 Institute of Pathology, Liebermeisterstraße 8, University of TübingenD-72076 Tübingen, Germany; 3 Institute of Anatomy, Oesterbergstraße 3, University of TübingenTübingen, Germany; 4 Department of Molecular Physiology and Biophysics, Department of Neurology, and Department of Internal Medicine, Howard Hughes Medical Institute, Carver College of Medicine, The University of IowaIowa City, IA, USA

**Keywords:** aquaporin-4, astrocytes, blood–brain barrier, freeze-fracturing, mice, orthogonal arrays of particles

## Abstract

The dystrophin–dystroglycan complex (DDC) is a molecular array of proteins in muscle and brain cells. The central component of the DDC is dystroglycan, which comprises α- and β-subunits. α-Dystroglycan (α-DG) binds to extracellular matrix components such as agrin, whereas β-dystroglycan (β-DG) is a membrane-spanning protein linking α-DG to the cytoskeleton and other intracellular components such as α-syntrophin. In astrocytes, α-syntrophin binds to the water channel protein aquaporin-4 (AQP4). Recently, it has been shown that AQP4 expression is unaltered in agrin-knockout mice, but that formation of orthogonal arrays of particles (OAPs), consisting of AQP4, is abnormal. As the brain-selective deletion of the DG gene causes a disorganization of the astroglial endfeet, we investigated whether DG deletion has an impact on AQP4. Western blotting revealed reduced AQP4 in the parenchymal but not in the superficial compartment of the astrocyte-conditioned DG-knockout mouse brain. Accordingly, immunohistochemical stainings of AQP4 revealed a selective loss of AQP4 in perivascular but not in superficial astroglial endfeet. In both superficial and perivascular endfeet of the DG-knockout brain, we observed a loss of OAPs. We conclude that in the absence of DG the majority of superficial AQP4 molecules did not form OAPs, and that expression of AQP4 in perivascular endfeet is compromised. However, the decreased number of perivascular AQP4 molecules obviously did form a few OAPs, even in the absence of DG.

## Introduction

The mammalian blood–brain barrier (BBB) is mainly characterized by tight junction-interconnected endothelial cells, the surrounding basal lamina and astrocytic processes abutting the vascular wall. In the mature system, astrocytes are highly polarized – where the glial membrane domain contacts the blood vessel, it is crowded with orthogonal arrays of intramembranous particles (OAPs) which can be visualized by means of freeze-fracture electron microscopy. This technique allows visualization of the membrane architecture by means of membrane cleavage – due to the double layering of the membrane lipids the interior of the membrane is hydrophobic. Therefore, a quickly frozen membrane can easily be cleaved. Both fractured leaflets can be shadowed with evaporated platinum and carbon to produce two complementary replicas of the same membrane. There are two means by which to look at the fractured membrane – if we look from outside the cell onto the inner leaflet, we have to imagine the cytoplasm behind the replica. Therefore, this replica is called the protoplasmic fracture face or P-face. If we look from inside the cell onto the outer leaflet, we have to imagine the extracellular space behind the replica. Therefore, this replica is called the external fracture face or E-face. The OAPs of astrocytes were detected by Dermietzel (1973) and could, for decades, be investigated exclusively by freeze-fracturing. They are associated with the P-face, and thus with the inner leaflet of the membrane. These OAPs are now well known to consist of the water channel protein aquaporin-4 (AQP4; for a recent review, see Wolburg *et al.*, 2011). AQP4 occurs in several variants due to alternative splicing. The most important variants are the M1 isoform (323 amino acids long) and the M23 isoform (22 amino acids shorter at the N terminus than M1; Jung *et al.*, 1994). The M23 isoform exhibits a much greater water transport capacity than the M1 isoform (Silberstein *et al.*, 2004). Both isoforms elicit distinct OAP morphologies – when CHO-K1 cells are transfected with AQP4 M1, no or very small arrays are found in the membranes, whereas when AQP4 M23 is transfected, large raft-like lattices are formed. When both isoforms were expressed together, OAPs were formed that strongly resembled those typical for astrocytes *in vivo* (Furman *et al.*, 2003), suggesting that both isoforms coexist in a single array (Nicchia *et al.*, 2010).

Anti-AQP4-immunoreactivity is commonly restricted to astroglial endfeet at the superficial surface of the brain and at the glio-vascular border (Nico *et al.*, 2001). Despite sophisticated analysis of OAPs in highly resolved freeze-fracture replicas (Rash *et al.*, 2004) and unraveling the atomic structure of AQP4 by electron crystallography of double-layered, two-dimensional crystals (Hiroaki *et al.*, 2006; Tani *et al.*, 2009), there is no absolute certainty regarding the correspondence of structure and stoichiometry of AQP4/OAP. Regardless, the number of subunits per OAP varies between four and more than 100. At the molecular level, AQP4 is connected to α-syntrophin which is a member of the dystrophin–dystroglycan complex (DDC). Dystroglycan (DG), another member of the DDC, connects to dystrophin and the cytoskeleton inside the cell and to the extracellular matrix (ECM) outside the cell (for detailed depiction of this molecular arrangement, see Fig. 3 in Wolburg *et al.*, 2009b; Fig. 4 in Wolburg *et al.*, 2009a; and Fig. 6 in Noell *et al.*, 2009). Binding of the ECM proteoglycan agrin to α-dystroglycan (α-DG) has recently been found to facilitate the formation of OAPs from AQP4 molecules: Noell *et al.* (2009) observed a loss of OAPs in the agrin-knockout mouse, but no loss of the AQP4 protein, suggesting a strong influence of agrin on the assembly or stability of AQP4 to form OAPs.

**FIG. 3:**
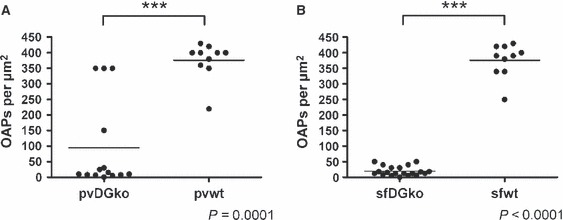
Statistical analysis for the comparison of OAP densities in the perivascular (A) and superficial (B) endfeet of the wild-type and DG-knockout mice was done by means of the Mann–Whitney test with graphpad software. pvDGko, perivascular endfeet in the DG-knockout; pvwt, perivascular endfeet in the wild-type; sfDGko, superficial endfeet in the DG-knockout; sfwt, superficial endfeet in the wild-type. Each dot represents one endfoot. In some perivascular endfeet of the DG-knockout mice, the variability of the OAP densities was substantial, as not only endfeet with low densities or no OAPs were found, but also endfeet with normal OAP densities. The variability of the wild-type endfeet was quite low. The horizontal bars correspond to the mean values. The difference between knockout and wild-type values is highly significant. *** indicates *P* = 0.0001.

**FIG. 4:**
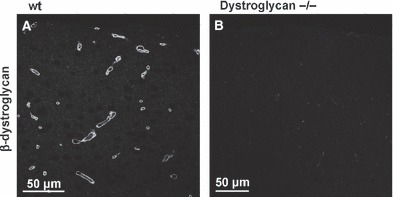
Immunoreactivity against β-DG of the brain of the wild type (A) and the GFAP-Cre/DG-null mouse (B). β-DG is present only in the wild-type mouse brain.

**FIG. 6:**
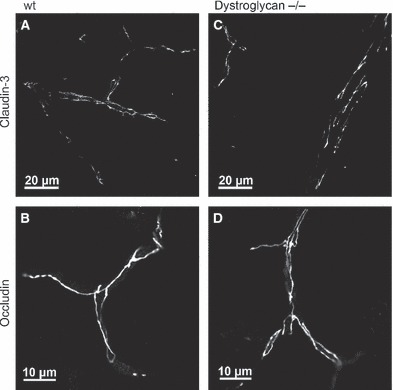
Immunoreactivity against tight junction proteins occludin and claudin-3 of the brain of the wild-type (A, B) and the GFAP-Cre/DG-null mouse (C, D). The tight junction proteins were equally present and distributed in both the wild-type and the GFAP-Cre/DG-deficient mouse brain microvessel endothelial cells.

Moore *et al.* (2002) crossed floxed DG mice with GFAP-cre mice to generate a conditional knockout of DG in brain. This brain-selective deletion of DG resulted in brain malformations and a disorganization of the astroglial endfeet structures. Therefore, we sought to determine the consequence of DG deletion on endfoot architecture and the expression and distribution of relevant molecules at the glial and endothelial surfaces.

## Materials and methods

### Animals

The generation and genotyping protocols for floxed-DG mice and brain-specific DG deletion (GFAP-cre/DAG1^loxneo^) mice have been described previously (Cohn *et al.*, 2002; Moore *et al.*, 2002). GFAP-cre/DAG1^loxneo^ or GFAP-cre/DAG1^lox^ (aka GFAP-cre/T30mN) mice were used for this study. The DAG1^loxneo^ allele contains two loxP sites flanking a neo selection cassette and a third loxP site downstream of exon 2 to enable DAG1 gene knockout. The DAG1^lox^ (aka T30mN) allele arose spontaneously from a Cre/DAG1^loxneo^ colony when a cre recombination event occurred involving only the loxP sites flanking neo to specifically remove the neo cassette. The DAG1^lox^ minus neo allele was transmitted germline and backcrossed to C57BL/6J for five generations. DAG1^lox^ mice were then mated to hemizygous GFAP-cre mice and knockouts were generated by L/L × cre, L/L breeding. GFAP-cre/DAG1^loxneo^ and GFAP-cre/DAG1^lox^ are phenotypically undistinguishable (K.P.C. laboratory, unpublished data). Experimental mice were identified by PCR genotyping of tail DNA. Mice were killed at 8 weeks of age for tissue, as described below. All animal procedures were approved by the IACUC committee at the University of Iowa.

### Freeze-fracture experiments

Mice were deeply anesthetized and transcardially perfused with 2.5% glutaraldehyde in 0.1 m cacodylate buffer (pH 7.4), and the brains were postfixed in the same fixative for 2 h at room temperature in the laboratory of K.P.C. Tissues were shipped within cacodylate buffer to the laboratory of H.W. The specimens were cryoprotected for freeze-fracturing in 30% glycerol and snap-frozen in nitrogen slush (−210 °C). Subsequently, they were fractured in a Balzer’s freeze-fracture device (BAF400D; Balzers, Liechtenstein) at 5 × 10^−6^ hPa and −150°C. The fracture faces were shadowed with platinum/carbon (3 nm, 45°) for contrast and carbon (30 nm, 90°) for stabilization of the replica. After removal of the cell material in 12% sodium hypochlorite, the replicas were rinsed in double-distilled water several times and mounted on Pioloform-coated copper grids. The replicas were observed by using a Zeiss EM10 electron microscope (Zeiss, Oberkochen, Germany). A scatter plot for evaluation of the OAP densities was performed using graphpad software (San Diego, CA, USA; http://www.GraphPad.com).

### Antibodies

The following antibodies were used to detect specific water channel and tight junction proteins: polyclonal rabbit anti-aquaporin-4 antiserum (Santa Cruz, Heidelberg, Germany), polyclonal rabbit anti-agrin (kindly provided by S. Kröger, Institute of Physiology, LMU Munich, Germany), monoclonal anti-agrin (Thermo Scientific, Rochford, MA, USA), polyclonal rabbit anti-claudin-3 (Zymed, San Francisco, CA, USA), polyclonal rabbit anti-occludin (Zymed), polyclonal anti-GFAP (DAKO, Hamburg, Germany), mouse monoclonal α-dystroglycan (clone VIA 4-1; Millipore, Schwalbach, Germany) and mouse monoclonal β-dystroglycan (Novocastra, Newcastle, UK). All antisera were used at a dilution of 1 : 100, with the exception of anti-GFAP (1 : 500) and α-DG (1 : 20).

The secondary goat anti-mouse and anti-rabbit antibodies labeled with cyanin-derivative dye Cy3 or Cy2 were purchased from Dianova (Hamburg, Germany). For controls, the primary antibody was omitted.

### Immunocytochemistry

Following cervical dislocation, mouse brains were removed and cut into six pieces to enhance post-fixation in 10% neutral buffered formalin. The tissue was embedded in paraffin and sectioned at 3 μm using a microtome (HM355SS; Micron international, Walldorf, Germany). Sections were placed on Super Frost Plus slides (Micron international), dewaxed, and rehydrated by descending alcohol concentrations to distilled water. For antigen retrieval, they were heated in a steamer in citrate buffer, pH 6.0, for 4 min, and coated in TBS buffer. To avoid non-specific staining, the sections were blocked by incubation with 5% (w/v) skimmed milk powder, 0.03% (v/v) Triton X-100 (Serva, Heidelberg, Germany) and 0.4% (w/v) NaN_3_ in TBS for 30 min. Primary antibodies diluted in the same solution were applied overnight at 4 °C. After three washes in TBS for 10 min, sections were incubated for 45 min with the secondary antibody at room temperature. Following washes in TBS, sections were mounted in Mowiol (Calbiochem, Merck, Germany). Sections were analysed with a confocal laser scanning microscope (LSM510 META with an Axioplan 2 microscope stand; Zeiss, Göttingen/Jena, Germany) using lasers at 488, 546 and 633 nm for excitation with appropriate filter sets. The system’s multi-track function was used to generate images for each stain and excitation sequentially. Images were processed using Adobe Photoshop (version 7.0; Adobe, Mountain View, CA, USA).

For staining tight junction proteins, cryosections of unfixed brains were briefly fixed for 10 min in ice-cold ethanol/acetone and then processed for immunostaining as described above.

As a control for non-specific staining or autofluorescence, the primary antibody was omitted. Non-specific binding was blocked by incubation for 30 min in 4% normal goat serum and 1% BSA in PBS. Specimens were mounted in Mowiol (Calbiochem). Fluorescence was visualized with a LSM510 META confocal laser scanning microscope (Zeiss) using a 40 × oil-immersion objective lens (N.A. 1.3) and a HeNe laser for excitation at 543 nm with appropriate filter sets.

### Electrophoresis and -immunoblotting

Brain tissue of wild-type and DG-knockout mice were lysed and prepared for Western blotting as described by Neely *et al.* (1999). Briefly, tissue was lysed with Laemmli-buffer, and protein was measured using the method of Bradford (1976). Five micrograms of total protein of each sample was used for electrophoresis on a 12.5% SDS-PAGE gel. The samples were blotted on a nitrocellulose membrane and stained with an antibody against AQP4 (Santa Cruz) and a secondary antibody labeled with horseradish peroxidase (Sigma, Deisenhofen, Germany). Western blots were densitometrically quantified using imagej software (NIH, Bethesda, MD, USA;http://rsb.info.nih.gov/ij). Absolute optical density (OD) was normalized to the ODs of the corresponding bands of β-tubulin loading control and expressed as relative abundance in arbitrary units. Each experiment was performed at least nine times. The statistical analysis for comparison of the experimental groups was carried out by Kruskal–Wallis one-way anova on ranks (sigma plot Software, Systat Software, San Jose, CA, USA; available at http://www.sigmaplot.com/). For posthoc pair wise comparison the Tukey–Kramer test was used.

## Results

### Freeze-fracturing

**FIG. 1:**
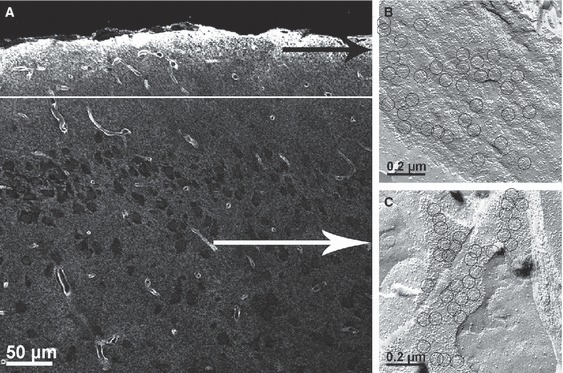
(A) Immunoreactivity of AQP4 in the wild-type mouse brain. Both the superficial and the perivascular astroglial endfeet were strongly positive for AQP4. Arrows point to regions of the brain from which the freeze-fracture replicas B and C were taken. (B) Freeze-fracture replica from superficial astroglial endfeet showing many OAPs (most of them, but not all, are encircled). The OAP density of this endfoot is of the order of 100/μm^2^. (C) Freeze-fracture replica from perivascular astroglial endfeet with many OAPs, the density again being of the order of 100/μm^2^.

**FIG. 2:**
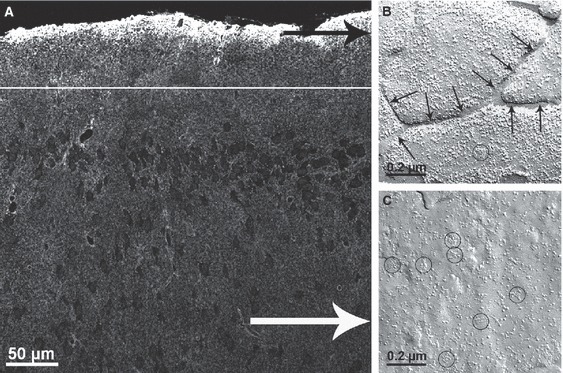
(A) Immunoreactivity of AQP4 in the GFAP-Cre/DG-null mouse brain. In contrast to the perivascular astroglial endfeet, only the superficial endfeet were strongly positive for AQP4. Arrows point to regions of the brain from which the freeze-fracture replicas B and C were taken. (B) Freeze-fracture replica from superficial astroglial endfeet showing only one single OAP (encircled). The OAP density of this endfoot is of the order of < 1/μm^2^. The border of the endfoot is demarcated by small arrows. (C) Freeze-fracture replica from perivascular astroglial endfeet with few OAPs, the density being of the order of < 10/μm^2^.

We performed freeze-fracture analyses of the astroglial endfeet in one wild-type and two DG-knockout mice. Superficial endfeet were identified as associated with the subpial space and meningeal cells; perivascular endfeet were identified in freeze-fracture replicas based on adjacency to endothelial cells (Fig. 1). In the wild-type mouse, density of OAPs was as expected (Wolburg, 1995) in the range 350/μm^2^ in both superficial and perivascular endfeet (Fig. 1B and C). In both types of endfeet from DG-knockout mice, there was greater variability of OAP densities compared with wild-types. In some superficial endfeet, we observed a nearly normal density of OAPs (more than 200/μm^2^), whereas in other endfeet there was a substantial loss of OAPs in both types of endfeet. Near or total loss of OAPs was also found: we observed endfeet that only contained one single OAP (Fig. 2B). In a membrane adjacent to that shown in Fig. 2B there were no OAPs. This membrane belonged to a neighboring endfoot, which could be identified by their typical inter-endfeet grooves (arrows in Fig. 2B). These grooves indicated the curvature of the membrane from the plane of the endfoot into the deeper regions of the sub-endfoot environment. Figure 3 shows a graph with original counting data from perivascular and superficial endfeet of wild-type and DG-knockout mice. Remarkably, the variability of OAP density in the wild-type mice was quite low. In summary, in the DG-knockout mouse we observed a loss, but not disappearance, of OAPs in both types of endfeet.

### Immunohistochemistry

AQP4-immunoreactivity of the wild-type mouse brain was as expected and restricted to the perivascular astrocytic endfeet (Fig. 1A). Although non-endfoot membrane domains are also known to contain a small number of AQP4 water channels, immunohistochemistry was not sensitive enough for their detection. Therefore, immunolabeling was used as a specific labeling of the blood vessels. At the surface of the brain, there was a broad zone of AQP4-positive immunoreactivity and no restriction to one endfoot membrane (Fig. 1A, above the white line). However, this was in accordance with the observation of Noell *et al.* (2009) which located AQP4 in a multilayered band of astroglial processes in the wild-type mouse.

In the GFAP-Cre/DG-null mouse, the superficial immunoreactivity was unaltered in comparison with the wild-type. Again, astroglial processes formed a multilayered meshwork several microns deep in the surface of the cerebral cortex (Fig. 2A, above the white line). However, and in sharp contrast to AQP4-immunoreactivity in the wild-type brain, perivascular endfeet staining was extremely weak (Fig. 2A, below the white line). Although it is difficult to quantify immunofluorescent signals, there was an obvious difference in the detection level between perivascular endfeet of wild-type and DG-deficient mice (compare Figs 1A and 2A).

**FIG. 5:**
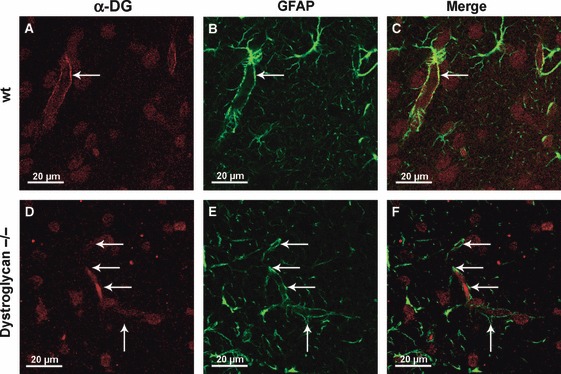
Immunoreactivity against α-DG (red) and GFAP (green) of the brain of the wild-type (A–C) and the GFAP-Cre/DG-null mouse (D–F). α-DG is present only in the wild-type mouse brain and co-localizes with GFAP-positive astrocytes. The arrows in A, B and C point to a doubled-labeled astrocytic endfoot membrane, which is presented in C in the merged colour yellow. The arrows in D, E and F point to an endfoot only stained by the astrocytic GFAP in green (E) and devoid of astrocytic DG (D).

We confirmed the presence or absence of dystroglycan in the wild-type and the brain-selective DG-knockout mice (Moore *et al.*, 2002), respectively. As expected, in wild-type brains, β-dystroglycan (β-DG) immunoreactivity was restricted to the astrocytic perivascular endfeet (Fig. 4A) and, to the same extent, superficial endfeet (data not shown), whereas immunoreactivity was not detectable in DG-knockout brains (Fig. 4B). In co-labeling experiments for GFAP and α-DG, we found distinct immunoreactivity in the glial endfeet membranes in wild-type (Fig. 5A–C) but no α-DG staining in the endfeet of knockout mice (Fig. 5D–F).

In summary, we observed a selective loss of AQP4 in perivascular but not in superficial astroglial endfeet of DG-knockout mice. Thus, the deficiency of DG compromises selectively the expression of AQP4 in the perivascular endfeet. In combination with the freeze-fracture results, we have shown that in the superficial endfeet DG plays a pivotal role in the formation of OAPs. In the perivascular endfeet of the DG-knockout mice, expression or membrane insertion of AQP4 is hindered, explaining the reduction of OAPs in the endfeet membranes.

The intact BBB is presumed to be constitutively associated with highly polarized astrocytes. This implies that a reduced astroglial polarity should compromise the quality of the BBB including the composition of the endothelial tight junctions. To assess endothelial tight junctions, we tested the presence and distribution of the tight junction proteins occludin, claudin-3, claudin-, and ZO1. We did not detect any differences in the expression patterns of these proteins (Fig. 6; data not shown for claudin-5 or ZO1).

**FIG. 7:**
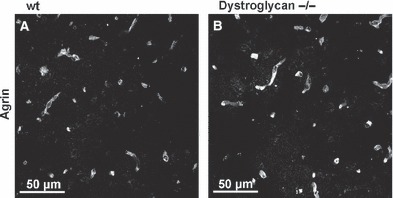
Immunoreactivity against agrin of the brain of the wild-type (A) and the GFAP-Cre/DG-null mouse (B). Again, agrin was equally present and distributed around both the wild-type and the GFAP-Cre/DG-deficient mouse brain microvessel endothelial cells.

Finally, our observations that the extracellular heparansulfate proteoglycan agrin influences the localization of AQP4 (Noell *et al.*, 2009), that α-DG influences AQP4 and that agrin is a known binding partner of α-DG prompted us to test the distribution and expression of agrin in wild-type and DG-knockout brains. However, we did not observe any difference in the agrin expression and distribution between wild-type and DG-knockout mice (Fig. 7).

### Western blotting

**FIG. 8:**
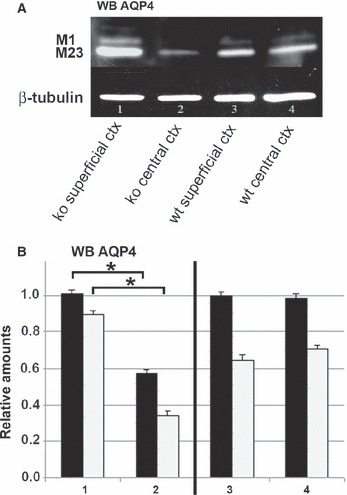
Western blots and relative amounts of AQP4 protein of superficial and deeper (central or parenchymal) cortical layers (superficial and central cortex) in DG-knockout (lanes/paired columns 1 and 2 in A and B), and in the wild-type mice (lanes/paired columns 3 and 4 in A and B). The upper bands in A and the white bars in B represent the AQP4 isoform M1, the lower bands in A and the black bars in B the AQP4 isoform M23, which is consistently more pronounced. (A) Original Western blots; (B) semiquantitative analysis of the intensity of the Western blot bands (from nine experiments). The superficial cortex of the knockout/wild-type was compared with the deeper layers of the cortex (so-called ‘central’ cortex) (1 with 2, and 3 with 4, respectively), not the knockout cortex (1) with the wild-type cortex (3). Therefore, the graph was divided by a bar into two halves. For statistical tools used for the evaluation of the Western blots, see Materials and methods. There is a significant difference between M23 in superficial and central cortex, and between M1 in superficial and central cortex of the knockout (*P < 0.001), but no significant difference between M23 in superficial and central cortex, and between M1 in superficial and central cortex of the wild-type.

Due to the immunohistochemical observation that perivascular endfeet but not superficial endfeet showed reduced AQP4-immunoreactivity in the GFAP-Cre/DG-null brain, we performed Western blots of selected superficial and parenchymal/central brain tissues. Indeed, separate analysis of these brain regions revealed a much stronger Western blot signal from superficial than from parenchymal/central brain, confirming a reduced expression of AQP4 in the parenchymal tissue of the DG-null mice (compare Fig. 8A, lanes 1 and 2, and Fig. 8B, left half, *P* < 0.001). In the wild-type brain, there was no significant difference between superficial and parenchymal tissue (Fig. 8A, lanes 3 and 4, and Fig. 8B, right half). Western blotting was performed with an AQP4 antibody and showed two distinct bands, the lower band (32 kDa) representing the AQP4-M23 isoform (black columns in Fig. 8B) and the upper band (34 kDa) representing the AQP4-M1 isoform (white columns in Fig. 8B). The Western blot showed a stronger immunoreactivity for AQP4-M23 than for AQP4-M1 regardless of the genetic background of the tested mice.

## Discussion

It is now generally accepted that in the intact BBB the endothelial tight junctions and the basal lamina, together with the associated pericytes and surrounding astrocytes, are responsible for the implementation of barrier functions. Astrocytes send processes to blood vessels and to the surface of the brain forming endfoot-like structures (Mathiisen *et al.*, 2010) with distinct polarity: where the glial membrane contacts the basal lamina, its constitution is completely different from that compartment not in contact with the basal lamina, but with other glial cells or neuronal elements. This topological principle alone suggests that the basal lamina with its ECM components might be responsible for the implementation of the astroglial polarity. The astroglial membrane in contact with the basal lamina contains K^+^ and water channels, which are essential for the ionic homeostasis of the extracellular microenvironment of the brain.

The ECM component agrin plays a pivotal role in configuring the astroglial endfoot membrane. In the agrin-deficient mouse, Noell *et al.* (2009) demonstrated a substantial loss of OAPs in the endfeet membranes, without loss of AQP4. This suggests that agrin, *in vivo* and *in vitro*, may facilitate the clustering of AQP4 into OAPs (Noell *et al.*, 2007; Fallier-Becker *et al.*, 2011). If agrin is able to influence the molecular configuration of AQP4 in the endfoot membrane, one might presume that agrin binds to AQP4; but this is not the case. Rather, agrin and other ECM components bind to α-DG (Gee *et al.*, 1994). α-DG, in turn, is a member of the DDC, which connects to AQP4 via its PDZ-mediated association with α-syntrophin (Neely *et al.*, 2001). DG is a central molecule directed to both the extracellular space and to the cytoplasmic cytoskeleton. A role for the DDC as a transmembrane link between laminin and the actin cytoskeleton has been described by Ervasti & Campbell (1993); however, the effect of DG deficiency on the actin cytoskeleton has not been studied yet. We cannot exclude that altered links to the cytoskeleton may also contribute to the disturbed formation of OAPs.

Notably, we find here that DG deficiency disrupts the membrane architecture of the astroglial endfeet. In both types of endfeet, an (incomplete) loss of OAPs was observed. However, in the perivascular endfeet of the deeper layers of the cortex (the so-called ‘central’ cortex, Fig. 8) only, this loss was paralleled by a loss of AQP4 as shown by immunohistochemistry. Based on these data, we conclude that DG in the BBB-relevant perivascular endfeet is in some way responsible for the maintenance of AQP4 expression or OAP stability. In the original brain-specific DG-knockout study, Moore *et al.* (2002) observed, in addition to DG loss, an absence of dystrophin in the brain. This was explained by a disruption of the DDC. Here, the mechanism of AQP4 loss in DG-deficient astrocytes might also result from DDC disruption, but it remains to be clarified whether the AQP4 reduction is due to down-regulation or to degradation. It should be stressed that a minor population of OAPs were maintained in the membrane despite absence of DG, and this was true for both types of endfeet.

The loss of OAPs in the superficial astrocytic endfeet of the DG-deficient mouse was not paralleled by a reduction in the immunohistochemical staining for AQP4. In contrast to the perivascular endfeet, AQP4-immunoreactivity did not reveal any difference to the wild-type mouse. This may suggest that DG at the superficial endfoot has no influence on the maintenance of AQP4 expression, but rather on the clustering of AQP4 molecules into OAPs. This means that, at least under these conditions of DG absence, AQP4 can exist as non-OAPs. The possible argument that the AQP4 isoform M1 does not form OAPs (Furman *et al.*, 2003) and that therefore the disappearance of most OAPs could be explained by a switch to the M1 isoform, cannot be applied here, because we find, by means of Western blotting, an unaltered M1/M23 ratio in which the amount of M23 steadily exceeds that of M1.

Interestingly, Nicchia *et al.* (2008) postulated that AQP4 pools differ by means of their dependency on dystrophin. The authors used two different dystrophin-deficient mice strains, the DP71-knockout which lacks the glial dystrophin gene product, and mdx3cv mice which show a drastic reduction of all dystrophin isoforms. The authors identified a large-molecular-weight AQP4 pool (> 1 MDa) dependent on dystrophin localized in perivascular astrocytes, and a smaller-molecular-weight AQP4 pool (500 kDa) independent of dystrophin localized in the granular cell layer of the cerebellum and in the subpial astroglial endfeet and ependymal cells. This finding suggests that the composition of AQP4 pools in glial membranes concerned with the management of the BBB is under the control of dystrophin, in contrast to other (subpial) membrane domains not involved in the BBB. As dystrophin as well as DG are members of the DDC, we might expect that AQP4 at the BBB is similarly dependent on DG. Nevertheless, here we show a dependence of AQP4 clustering in the superficial and of AQP4 presence in the perivascular endfeet on DG.

Nicchia *et al.* (2010) postulated, on the basis of blue native SDS-PAGE analysis of a variety of organs that the differently sized AQP4 pools differ in their M1/M23 ratio by enrichment of M1 in the low-molecular-weight and M23 in the high-molecular-weight AQP4 pool. This would mean that subpial/perivascular astroglial endfeet would be dominated by an increased/decreased M1/M23 ratio, respectively, and that M23 more than M1 would be of high significance for the stability of the BBB. This conclusion cannot be confirmed from our results which clearly reveal a stable M1/M23 ratio in wild-type and DG-deficient mice. On the other hand, there must be a functional difference between the superficial and perivascular endfeet, because the perivascular but not the superficial glial processes are under the imminent influence of the endothelial basal lamina.

If the DG-deficient mouse suffers from a loss of AQP4 in perivascular astroglial endfeet, then we need to ask whether the phenotype of the glio-vascular complex resembles that of the AQP4-knockout mouse as described by Manley *et al.* (2000). In contrast to wild-type animals, this mutant is resistant to water intoxication. One could assume that the DG-deficient mouse, due to the loss of perivascular AQP4, might similarly survive such water intoxication or ischemic stroke. However, this was not tested.

With regard to OAP-related polarity of astrocytes and the quality of the BBB, Zhou *et al.* (2008) found an altered BBB integrity with serious increase of permeability and downregulation of the astrocyte-specific intermediate filament GFAP in the AQP4-knockout mouse. In contrast, Saadoun *et al.* (2009) found no difference in wild-type and AQP4-knockout mice concerning the integrity of the BBB. Similarly, we find no evidence here of any impairment of the BBB: the tight junction molecules ZO-1, occludin and claudin-5 were unaltered in the DG-knockout mouse (Fig. 6B and D). The regulation and control of the BBB is so complex and finely tuned that it seems improbable that altered expression of one BBB-relevant molecule can singularly impair the BBB. Rather, any disturbance of the BBB caused by a single factor should be counter regulated, suggesting that barrier disturbance including edema formation and clinical imaging of increased permeability is the result of a number of disrupting factors.

In conclusion, we have shown for the first time that DG is necessary for the formation of the glial endfoot architecture. Together with agrin, DG is responsible for the glial polarity and thus for a functional BBB. The detailed function of the ECM–DDC complex in different astroglial processes must be elucidated further to better understand BBB regulation.

## Abbreviations

α-DG: α-dystroglycan
β-DG: β-dystroglycan
AQP4: aquaporin-4
BBB: blood–brain barrier
DDC: dystrophin–dystroglycan complex
DG: dystroglycan
ECM: extracellular matrix
OAPs: orthogonal arrays of particles
OD: optical density
